# Maternal Cardiac Disease and Congenital Heart Disease Risk in Offspring

**DOI:** 10.1001/jamanetworkopen.2026.10823

**Published:** 2026-05-05

**Authors:** Yanji Qu, Xiaoqing Liu, Shao Lin, Michael S. Bloom, Yanli Liu, Haiyun Yuan, Ximeng Wang, Bingjun Huang, Mayank Dalakoti, Xiangmin Gao, Yong Wu, Xinli Zhou, Jian Zhuang, Jimei Chen, Jie Li

**Affiliations:** 1 Department of Cardiac Surgery, Guangdong Cardiovascular Institute, Guangdong Provincial People’s Hospital, Guangdong Academy of Medical Sciences, Southern Medical University, Guangzhou, Guangdong, China; 2 Global Health Research Center, Guangdong Provincial People’s Hospital, Guangdong Academy of Medical Sciences, Southern Medical University, Guangzhou, Guangdong, China; 3 Department of Environmental Health Sciences, University at Albany State University of New York, Rensselaer, Albany; 4 Department of Global and Community Health, George Mason University, Fairfax, Virginia; 5 Department of Obstetrics and Gynecology, Guangdong Provincial People’s Hospital, Guangdong Academy of Medical Sciences, Southern Medical University, Guangzhou, Guangdong, China; 6 British Heart Foundation Cardiovascular Epidemiology Unit, Department of Public Health and Primary Care, University of Cambridge, Cambridge, United Kingdom; 7 Cardiovascular Metabolic Translational Research Program, National University of Singapore, Singapore; 8 Department of Cardiology, National University Heart Centre Singapore, Singapore; 9 Department of Epidemiology and Center for Global Cardiometabolic Health, School of Public Health, Brown University, Providence, Rhode Island

## Abstract

**Importance:**

Maternal adult congenital heart disease (ACHD) has been associated with increased offspring congenital heart disease (CHD), but evidence from resource-limited regions remains scarce. The association between maternal acquired heart disease (AHD) and offspring CHD is unknown.

**Objective:**

To quantify the overall and subtype-specific CHD risk in offspring associated with maternal ACHD and AHD, examine the association of maternal ACHD and AHD with outcomes in offspring with CHD, and identify maternal factors that may modify the associations between maternal cardiac diseases and offspring CHD risk.

**Design, Setting, and Participants:**

This prospective birth cohort study enrolled pregnant women receiving prenatal care between August 1, 2011, and December 31, 2021, at a major cardiac referral center in China. Participants included pregnant women with ACHD, with AHD, or without cardiac disease and were followed up through delivery; their offspring were followed up until 1 year of age. All follow-ups were completed by December 15, 2023. Data were analyzed from April 1, 2024, through April 31, 2025.

**Exposures:**

Maternal ACHD and AHD, confirmed via the center’s electronic medical records.

**Main Outcomes and Measures:**

The main outcome was offspring CHD, which was diagnosed using echocardiography. Log-binomial regression was used to estimate relative risks (risk ratios [RRs]) and 95% CIs. Adverse outcomes were compared using pairwise tests. Stratification analyses identified potential effect modifiers.

**Results:**

A total of 14 336 pregnant women with 15 677 offspring (8480 males [54.1%]) were included. The mean (SD) maternal age and gestational age at enrollment were 31.4 (4.5) years and 16.4 (6.4) weeks, respectively. Both maternal ACHD and AHD were associated with higher CHD risk in offspring (RR, 1.71 [95% CI, 1.26-2.31] and 1.38 [95% CI, 1.02-1.87], respectively). Minor CHDs, particularly septal defects, were the subtypes with the greatest magnitude of associations with maternal ACHD (RR, 2.95; 95% CI, 1.97-4.43) and AHD (RR, 2.28; 95% CI, 1.50-3.45). Right ventricular outflow tract obstruction (RR, 6.17; 95% CI, 3.59-10.60) and valvular heart disease (RR, 1.65; 95% CI, 1.11-2.45) were the key contributors to offspring CHD risk. Preterm birth had higher rates among offspring with CHD and mothers with ACHD as well as offspring with CHD and mothers without ACHD compared with offspring without CHD and mothers without cardiac disease (12 of 39 [30.8%] and 121 of 780 [15.5%] vs 1287 of 14 088 [9.1%]; all *P* < .001). Higher rates of chromosomal (5 of 39 [12.8%] and 38 of 780 [4.9%] vs 75 of 14 088 [0.5%]; all *P* < .001) and genetic aberrations (3 of 39 [7.7%] and 16 of 780 [2.1%] vs 57/14 088 [0.4%]; all *P* < .001) were found among offspring with CHD and mothers with AHD as well as offspring with CHD and mothers without AHCD compared with offspring without CHD and mothers without cardiac disease. Associations between maternal cardiac disease and offspring CHD were robust in primiparous women (ACHD: RR, 2.15 [95% CI, 1.48-3.11], *P* for interaction < .001; AHD: RR, 1.73 [95% CI, 1.17-2.56], *P* for interaction = .02) and those with periconceptional exposure to hazardous substances (ACHD: RR, 2.22 [95% CI, 1.56-3.16], *P* for interaction < .001; AHD: RR, 1.57 [95% CI, 1.05-2.36], *P* for interaction = .02).

**Conclusions and Relevance:**

In this cohort study, maternal ACHD and AHD were associated with increased risks and adverse outcomes of offspring CHD. Targeted modification of identified maternal factors could help mitigate offspring CHD risk in this high-risk population.

## Key Points

**Question:**

What is the association of maternal adult congenital heart disease (ACHD) and acquired heart disease (AHD) with the risk and outcomes of congenital heart disease (CHD) in offspring?

**Findings:**

In this cohort study of 14 336 pregnant women with 15 677 offspring in China, maternal ACHD and AHD were significantly associated with higher risks of offspring CHD, particularly septal defects. Among offspring with CHD, maternal ACHD was associated with higher rates of preterm births, whereas maternal AHD was associated with increased chromosomal and genetic abnormalities.

**Meaning:**

The finding of an association between maternal cardiac disease and elevated offspring CHD risk and adverse outcomes supports targeted screening and management in this high-risk population of pregnant women.

## Teaser

This cohort study examines the risk of congenital heart disease in offspring of women with adult congenital heart disease and acquired heart disease living in a resource-limited setting in China.

## Introduction

Congenital heart disease (CHD) is the most common birth defect globally, affecting approximately 9.1 per 1000 live births.^1^ CHD is the leading contributor to infant and childhood deaths related to birth defects, accounting for 30% to 50% of mortality.^2,3^ Meanwhile, advances in pediatric cardiac care have substantially improved survival, allowing more children with CHD to reach adulthood.^4^ The prevalence of severe adult CHD (ACHD) increased by 85% from 1985 to 2000 and by 55% from 2000 to 2010.^5,6^ However, these adults remain at elevated risk for lifelong complications, such as heart failure, arrhythmia, pulmonary hypertension, and residual valvular lesions.^7^ Pregnancy can exacerbate these conditions, posing substantial dual risks for both mother and fetus.^8^

In parallel, acquired heart disease (AHD) during pregnancy is becoming increasingly prevalent due to older maternal age and higher rates of obesity, diabetes, and hypertensive disorders.^9^ Both ACHD and AHD are well-recognized risk factors for adverse maternal and neonatal outcomes, including preterm birth, fetal growth restriction, and neonatal intensive care unit admission.^10,11,12^ Moreover, the intergenerational transmission of CHD has received growing clinical attention.^13^

Previous studies have reported a higher CHD incidence in offspring of mothers with ACHD, ranging from 3% to 17% depending on the defect subtype, compared with the approximately 1% baseline risk observed in the general population.^8,14,15,16,17,18,19^ However, most evidence comes from high-income regions, leaving the generalizability to low-income regions uncertain. Similar concerns have been raised regarding the risk that maternal AHD poses for offspring CHD, but robust data quantifying this risk remain scarce worldwide.^20^ Small sample sizes, lack of appropriate control groups, and incomplete information on CHD phenotypes or associated outcomes often constrained prior studies. Critical gaps also persist in understanding how modifiable maternal factors may affect these associations.

To address these knowledge gaps, we conducted a large, prospective birth cohort study at a high-resource cardiac referral center in China. We aimed to (1) quantify the overall and subtype-specific CHD risk in offspring associated with maternal ACHD and AHD, (2) examine the association of maternal ACHD and AHD with outcomes in offspring with CHD, and (3) identify maternal factors that may modify the associations between maternal cardiac diseases and offspring CHD risk.

## Methods

### Study Design and Participants

This prospective birth cohort study was conducted at Guangdong Provincial People’s Hospital, one of China’s largest cardiac referral centers. Pregnant women receiving prenatal care between August 1, 2011, and December 31, 2021, were invited to participate. The Guangdong Provincial People’s Hospital Ethics Committee approved the study. Written informed consent was obtained from each participant at enrollment. We complied with the Declaration of Helsinki^21^ and followed the Strengthening the Reporting of Observational Studies in Epidemiology (STROBE) reporting guideline.

The cohort comprised pregnant women with ACHD, with AHD, or without cardiac disease. All women were followed up throughout pregnancy. Appropriate disease management approaches were adopted from international guidelines.^22,23^ Their offspring were followed up until 1 year after birth to capture late-identified CHD. All follow-ups were completed by December 15, 2023.

### Offspring CHD Diagnosis

Offspring CHD was diagnosed and confirmed through a multistage approach, including fetal ultrasonography screening, fetal echocardiography, neonatal cardiac evaluations, postnatal echocardiographic confirmation, and follow-up assessments (eMethods 1 and eFigure 1 in Supplement 1). All CHD phenotypes were coded with the *International Statistical Classification of Diseases and Related Health Problems, Tenth Revision* (*ICD-10*) codes Q20.000 to Q28.000. CHD cases were further classified as isolated or associated (with concurrent chromosomal, genetic, or noncardiac anomalies), single or multiple (>1 lesion), and minor or critical (requiring intervention in the first year after birth) as well as by cause. For subtype analyses, phenotypes were grouped into septal or nonseptal defects to ensure sufficient statistical power.

### Maternal Cardiac Disease Diagnosis

Maternal ACHD and AHD status was extracted from the hospital’s electronic medical records using a standardized case report form and was double-checked during the face-to-face interviews with participants for detailed information. *ICD-10*–coded diagnoses, prior interventions, comorbidities, and medication use were reviewed. AHD was categorized as valvular heart disease (VHD) excluding rheumatic etiologies, rheumatic heart disease, cardiomyopathy, and other conditions. All women with ACHD or AHD underwent standardized echocardiography, electrocardiography, and New York Heart Association (NYHA) functional assessments.

### Maternal Cardiac Complications and Perinatal Adverse Outcomes

Maternal cardiac complications included prior cardiac interventions, hypertensive disorders (blood pressure, 140/90 mm Hg on 2 occasions), arrhythmia, pulmonary hypertension, signs of heart failure (NYHA classification ≥II), and postpartum hemorrhage (>500 mL vaginal or 1000 mL cesarean delivery or transfusion-requiring). Perinatal adverse outcomes included non–live births (termination and fetal or neonatal death), fetal growth restriction or small for gestational age, preterm birth (<37 weeks’ gestation), low birth weight (<2500 g), chromosomal or genetic abnormalities, and non–cardiac defects.

### Statistical Analysis

Data were analyzed from April 1, 2024, through April 31, 2025. We used log-binomial regression models to estimate relative risks (or risk ratios [RRs]) and 95% CIs for offspring CHD associated with maternal ACHD and AHD. Model 1 was unadjusted. Model 2 was adjusted for maternal sociodemographic characteristics, reproductive factors, pregnancy complications, and periconceptional exposures (eMethods 2 in Supplement 1). Similar models were applied for subtype-specific analyses. We used a Sankey diagram to visualize the spectrum of offspring CHD phenotypes corresponding to maternal ACHD and AHD subtypes. Sensitivity analyses excluded referral cases, nonsingleton births, siblings, and cases with patent foramen ovale to assess the robustness of the results.

Offspring were stratified into 4 mutually exclusive groups based on maternal cardiac disease status and CHD presence: offspring without CHD and mothers without cardiac disease (reference group), offspring without CHD and mothers with ACHD or AHD, offspring with CHD and mothers without cardiac disease, and offspring with CHD and mothers with ACHD or AHD. Overall χ^2^ test and pairwise comparisons were performed across these groups to assess the adverse outcomes associated with maternal cardiac disease.

Potential effect modifiers of the associations between maternal cardiac disease and offspring CHD were evaluated using stratified analyses and likelihood ratio tests.^24^ The maternal factors considered were sociodemographic characteristics, pregnancy complications, reproductive factors, and periconceptional exposures. A 2-sided *P* < .05 was considered statistically significant. Analyses were conducted using SPSS, version 27 (IBM), and R, version 4.3.2 (R Project for Statistical Computing).

## Results

### Baseline Characteristics

Of the 18 902 pregnant women invited to participate, 3796 declined, yielding a participation rate of 79.9%. We excluded 770 pregnant women with isolated arrhythmia in structurally normal hearts, leaving a final analytic sample of 14 336 pregnant women with 15 677 offspring (7197 females [45.9%], 8480 males [54.1%]) (eFigure 2 in Supplement 1). At enrollment, the mean (SD) maternal age was 31.4 (4.5) years and the mean (SD) gestational age was 16.4 (6.4) weeks. The mean (SD) follow-up time was 6.6 (2.5) months for women and 15.0 (3.0) months for the offspring after birth.

As shown in Table 1, 858 offspring were diagnosed with CHD, resulting in an overall incidence of 5.5%. CHD was more common among offspring of mothers with ACHD or AHD than mothers without these diagnoses (39 of 368 [10.6%] and 39 of 441 [8.8%] vs 780 of 14 868 [5.2%]). Higher CHD rates were observed among mothers with lower educational level (129 [9.2%]), unemployment (156 [9.9%]), multiparity (367 [6.5%]), prior stillbirth or congenital anomalies (39 [9.3%]), and abortion history (266 [6.3%]). Similarly, elevated CHD rates were noted in mothers with multiple gestations (71 [10.1%]); pregnancies conceived by in vitro fertilization and embryo transfer (66 [9.3%]); and pregnancies complicated by diabetes (184 [6.3%]), kidney disease (26 [8.1%]), or anemia (75 [7.4%]). Additionally, maternal prepregnancy overweight (118 [6.5%]), periconceptional alcohol use (11 [10.5%]), lack of folic acid supplementation (79 [6.8%]), unstable emotions (47 [8.7%]), and medication use (383 [6.6%]) were associated with higher CHD rates. Maternal baseline characteristics stratified by absence or presence of maternal cardiac disease are presented in eTable 1 in Supplement 1.

**Table 1.** Maternal Baseline Characteristics

Maternal characteristic	Offspring, No. (%)			*P* value
	Overall (N = 15 677)	With CHD (n = 858)	Without CHD (n = 14 819)	
Diagnosis of cardiac disease				
ACHD	368 (2.3)	39 (10.6)	329 (89.4)	<.001
AHD	441 (2.8)	39 (8.8)	402 (91.2)	
None	14 868 (94.8)	780 (5.2)	14 088 (94.8)	
Sociodemographic				
Age, mean (SD), y	31.4 (4.5)	31.4 (4.7)	31.4 (4.5)	.85
Educational level^a^				
≤High school	1402 (8.9)	129 (9.2)	1273 (90.8)	<.001
Senior high school	1759 (11.2)	135 (7.7)	1624 (92.3)	
College	10 859 (69.3)	531 (4.9)	10 328 (95.1)	
≥Master’s degree	1647 (10.5)	61 (3.7)	1586 (96.3)	
Household income per person per mo, ¥				
<3500	2308 (14.7)	137 (5.9)	2171 (94.1)	.29
≥3500	13 369 (85.3)	721 (5.4)	12 648 (94.6)	
Occupation				
Unemployed	1577 (10.1)	156 (9.9)	1421 (90.1)	<.001
Individual proprietor	1500 (9.6)	120 (8.0)	1380 (92.0)	
Industry worker	711 (4.5)	36 (5.1)	675 (94.9)	
Government servant and professional	6487 (41.4)	296 (4.6)	6191 (95.4)	
Managerial and commercial service	4712 (30.1)	207 (4.4)	4505 (95.6)	
Other^b^	690 (4.4)	43 (6.2)	647 (93.8)	
Migrant^c^				
Yes	1814 (11.6)	108 (6.0)	1706 (94.0)	.34
No	13 863 (88.4)	750 (5.4)	13 113 (94.6)	
Reproductive factors				
Nulliparity				
Yes	10 012(63.9)	491 (4.9)	9521 (95.1)	<.001
No	5665 (36.1)	367 (6.5)	5298 (93.5)	
Reproductive history of stillbirth or congenital malformations				
Yes	419 (2.7)	39 (9.3)	380 (90.7)	<.001
No	15 258 (97.3)	819 (5.4)	14 439 (94.6)	
Elective abortion history				
Yes	4190 (26.7)	266 (6.3)	3924 (93.7)	.004
No	11 487 (73.3)	592 (5.2)	10 895 (94.8)	
Spontaneous abortion history				
Yes	1340 (8.5)	90 (6.7)	1250 (93.3)	.04
No	14 337 (91.5)	768 (5.4)	13 569 (94.6)	
Multiple gestations				
Yes	705 (4.5)	71 (10.1)	634 (89.9)	<.001
No	14 972 (95.5)	787 (5.3)	14 185 (94.7)	
IVF-ET				
Yes	712 (4.5)	66 (9.3)	646 (90.7)	<.001
No	14 965 (95.5)	792 (5.3)	14 173 (94.7)	
Pregnancy complications				
Hypertensive disorders				
Yes	791 (5.0)	45 (5.7)	746 (94.3)	.78
No	14 886 (95.0)	813 (5.5)	14 073 (94.5)	
Diabetes^d^				
Yes	2905 (18.5)	184 (6.3)	2721 (93.7)	.02
No	12 772 (81.5)	674 (5.3)	12 098 (94.7)	
Kidney disease				
Yes	322 (2.1)	26 (8.1)	296 (91.9)	.04
No	15 355 (97.9)	832 (5.4)	14 523 (94.6)	
Anemia				
Yes	1017 (6.5)	75 (7.4)	942 (92.6)	.006
No	14 660 (93.5)	783 (5.3)	13 877 (94.7)	
Prepregnancy overweight				
Yes	1819 (11.6)	118 (6.5)	1701 (93.5)	.04
No	13 858 (88.4)	740 (5.3)	13 118 (94.7)	
Self-reported periconceptional exposures^e^				
Smoking^f^				
Yes	108 (0.7)	8 (7.4)	100 (92.6)	.38
No	15 569 (99.3)	850 (5.5)	14 719 (94.5)	
Passive smoking^g^				
Yes	4107 (26.2)	239 (5.8)	3868 (94.2)	.26
No	11 570 (73.8)	619 (5.4)	10 951 (94.6)	
Alcohol use^h^				
Yes	105 (0.7)	11 (10.5)	94 (89.5)	.02
No	15 572 (99.3)	847 (5.4)	14 725 (94.6)	
Folic acid supplementation				
Yes	14 514 (92.6)	779 (5.4)	13 735 (94.6)	.04
No	1163 (7.4)	79 (6.8)	1084 (93.2)	
Unstable emotions				
Yes	542 (3.5)	47 (8.7)	495 (91.3)	<.001
No	15 135 (96.5)	811 (5.4)	14 324 (94.6)	
Virus infection^i^				
Yes	3649 (23.3)	220 (6.0)	3429 (94.0)	.09
No	12 028 (76.7)	638 (5.3)	11 390 (94.7)	
Fever: temperature >38.5 °C				
Yes	1096 (7.0)	61 (5.6)	1035 (94.4)	.89
No	14 581 (93.0)	797 (5.5)	13 784 (94.5)	
Contraception medication use				
Yes	1055 (6.7)	63 (6.0)	992 (94.0)	.46
No	14 622 (93.3)	795 (5.4)	13 827 (94.6)	
Other medication use^j^				
Yes	5784 (36.9)	383 (6.6)	5401 (93.4)	<.001
No	9893 (63.1)	475 (4.8)	9418 (95.2)	
Living in rooms newly renovated within 6 mo				
Yes	1140 (7.3)	62 (5.4)	1078 (94.6)	.96
No	14 537 (92.7)	796 (5.5)	13 741 (94.5)	
Residential proximity to main roadway <50 m				
Yes	11 311 (72.2)	621 (5.5)	10 690 (94.5)	.88
No	4366 (27.8)	237 (5.4)	4129 (94.6)	
Hazardous substances contact^k^				
Yes	8889 (56.7)	491 (5.5)	8398 (94.5)	.75
No	6788 (43.3)	367 (5.4)	6421 (94.6)	
Residential proximity to waste disposal stations or chemical plants <1 km				
Yes	268 (1.7)	20 (7.5)	248 (92.5)	.15
No	15 409 (98.3)	838 (5.4)	14 571 (94.6)	

Abbreviations: ACHD, adult congenital heart disease; AHD, acquired heart disease; CHD, congenital heart disease; IVF-ET, in vitro fertilization and embryo transfer.

SI conversion factor: To convert yuan to US dollars, multiply by 6.95.

^a^ There were 10 missing values for educational level.

^b^ Other occupation includes occupations beyond the categories shown here.

^c^ Migrants are defined as people living and working outside of their country of origin.

^d^ Diabetes includes pregestational and gestational, type 1, and type 2 diabetes.

^e^ Periconceptional period spans 6 months before conception through the first trimester.

^f^ Smoking is defined as consumption of at least 1 cigarette per day, on average.

^g^ Passive smoking is defined as exposure to tobacco smoke at home, workplace, or both.

^h^ Alcohol use is defined as intake of at least 50 mL of alcoholic drink per day, on average.

^i^ Virus infection includes influenza, mumps, measles, rubella, chicken pox, hepatitis, or other infections.

^j^ Medication use includes use of Chinese medication or pharmaceutical chemicals except for contraception medication.

^k^ Hazardous substances contact is defined as exposure to noise, organic solvents, pesticides, paint, heavy metals, radiation, or other teratogens.

### Maternal ACHD and AHD

Maternal AHD was more common than ACHD (441 [54.5%] vs 368 [45.5%]) (eTable 2 in Supplement 1). VHD was the top AHD subtype and accounted for 209 AHD cases (47.4%). Septal defects were the most frequent maternal ACHD subtype (207 [56.3%]), with atrial septal defects (135 [36.7%]) being the top phenotype. All pregnant women with ACHD had isolated lesions, except for 6 (1.6%) who had Marfan syndrome.

### Offspring CHD Phenotypes

Among the 858 offspring with CHD, 851 (99.2%) were diagnosed prenatally. In 102 offspring (11.9%), CHD was associated with chromosomal or genetic abnormalities or noncardiac defects (eTable 3 in Supplement 1). A total of 560 offspring (65.3%) had multiple CHDs, and 298 (34.7%) had single lesions. Critical CHD and minor CHD were evenly distributed. Septal defects were the most common subtypes (345 [40.2%]), with atrial septal defects as the leading phenotype among 220 offspring (25.6%). Transposition of the great arteries was the most common critical CHD phenotype (74 [8.6%]).

### Maternal Cardiac Disease and Offspring CHD Risk

**Figure 1.**
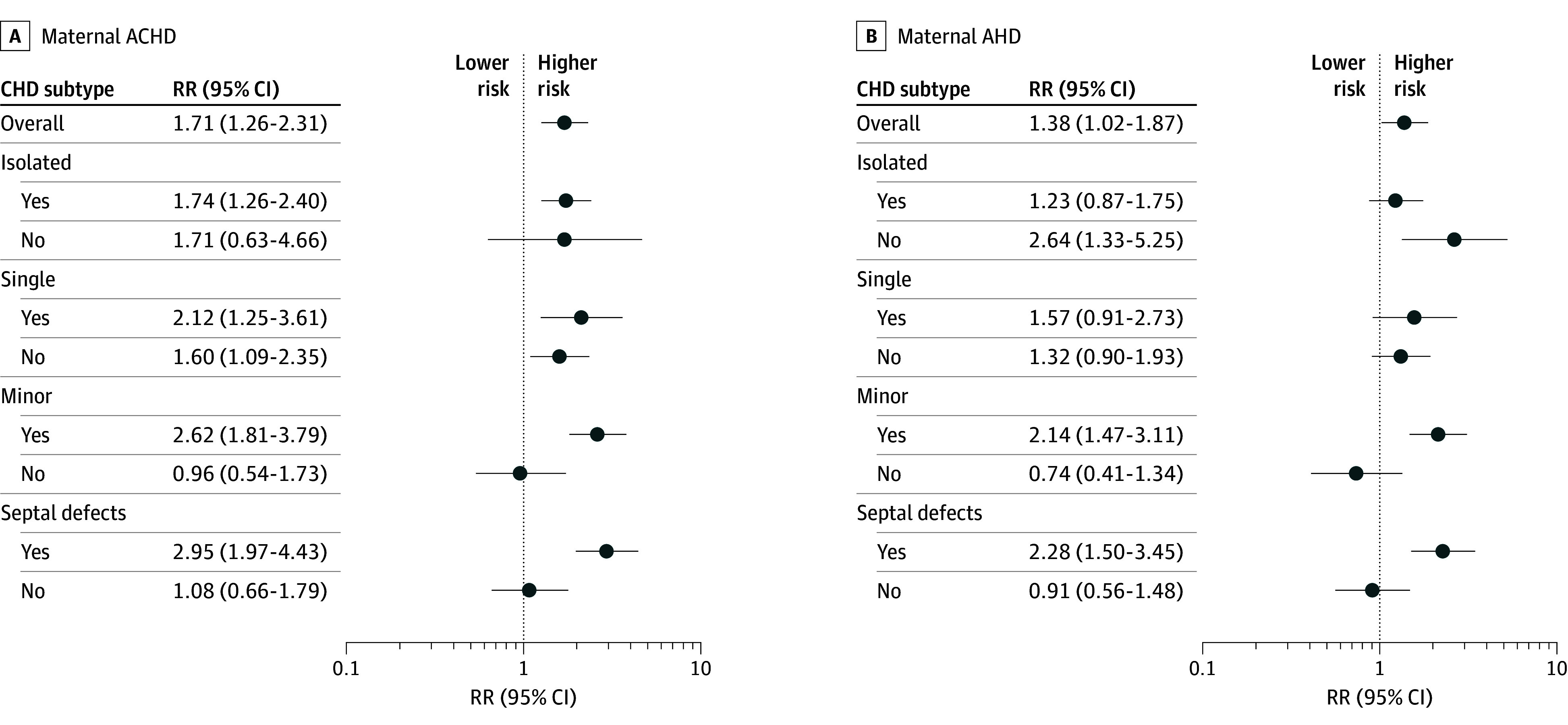
Dot Plots of Relative Risks of Offspring Congenital Heart Disease (CHD) Among Mothers With Adult Congenital Heart Disease (ACHD) and Acquired Heart Disease (AHD) Calculations adjusted for maternal sociodemographic characteristics (age at conception, educational level, and occupation), reproductive factors (nulliparity, multiple gestations, in vitro fertilization and embryo transfer, reproductive history of stillbirth or congenital malformations, and abortion history), pregnancy complications (hypertensive disorders, diabetes, kidney disease, anemia, and prepregnant overweight), and periconceptional exposures (smoking, alcohol use, folic acid supplementation, unstable emotions, and medicine use). Error bars represent 95% CIs. RR indicates risk ratio.

Both maternal ACHD and AHD were associated with increased risk of offspring CHD (RR, 1.71 [95% CI, 1.26-2.31] and 1.38 [95% CI, 1.02-1.87], respectively) (Figure 1; eTable 4 in Supplement 1). Sensitivity analyses restricted to nonreferral participants, singleton offspring, and singleton offspring from primiparous women or excluding patent foramen ovale cases showed similar or more robust results (eTable 5 in Supplement 1).

As shown in Figure 1 and eTable 4 in Supplement 1, minor CHDs—specifically septal defects—were the subtypes with the greatest magnitude of associations with maternal ACHD (RR, 2.95; 95% CI, 1.97-4.43) and AHD (RR, 2.28; 95% CI, 1.50-3.45). ACHD was primarily associated with isolated CHDs (RR, 1.74; 95% CI, 1.26-2.40), whereas AHD was linked to associated CHDs (RR, 2.64; 95% CI, 1.33-5.25).

**Figure 2.**
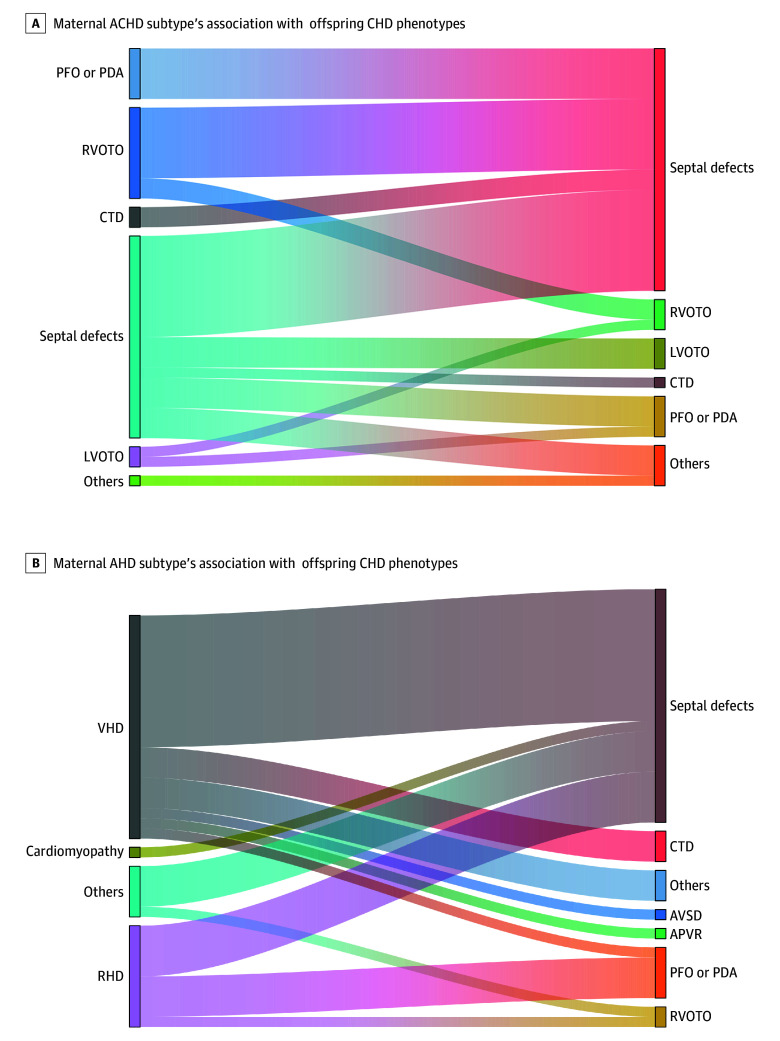
Sankey Diagram of the Spectrum of Offspring Congenital Heart Disease (CHD) Phenotypes Corresponding to Maternal Adult Congenital Heart Disease (ACHD) and Acquired Heart Disease (AHD) Subtypes APVR indicates anomalous pulmonary venous return; AVSD, atrioventricular septal defect; CTD, conotruncal defects; LVOTO, left ventricular outflow tract obstruction; PDA, patent ductus arteriosus; PFO, patent foramen ovale; RHD, rheumatic heart disease; RVOTO, right ventricular outflow tract obstruction; VHD, valvular heart disease, excluding rheumatic valvular anomaly.

By maternal ACHD subtype, right ventricular outflow tract obstruction (RVOTO) conferred the highest risk of offspring CHD (RR, 6.17; 95% CI, 3.59-10.60) (Table 2). Among maternal AHD subtypes, VHD presented the highest risk (RR, 1.65; 95% CI, 1.11-2.45). No distinct clustering of specific offspring CHD phenotypes was observed in relation to maternal subtypes (Figure 2).

**Table 2.** Relative Risk of Offspring Congenital Heart Disease by Major Maternal ACHD and AHD Subtypes

Maternal ACHD and AHD subtypes	Offspring, No.		CHD per 100 offspring	RR (95% CI)^a^		*P* value
	Total offspring	With CHD		Crude	Adjusted^b^	
ACHD	368	39	10.6	2.02 (1.49-2.74)	1.71 (1.26-2.31)	<.001
CTD	27	2	7.4	1.41 (0.37-5.37)	1.26 (0.34-4.76)	.73
LVOTO	14	2	14.3	2.72 (0.75-9.84)	2.32 (0.65-8.25)	.20
RVOTO	32	9	28.1	5.36 (3.07-9.37)	6.17 (3.59-10.60)	<.001^c^
Septal defects	207	20	9.7	1.84 (1.21-2.81)	1.48 (0.97-2.24)	.07
PFO or PDA	40	5	12.5	2.38 (1.05-5.43)	1.81 (0.81-4.05)	.15
AHD	441	39	8.8	1.69 (1.24-2.29)	1.38 (1.02-1.87)	.04
VHD^d^	209	22	10.5	2.01 (1.34-3.00)	1.65 (1.11-2.45)	.01^c^
RHD	173	10	5.8	1.10 (0.60-2.02)	0.84 (0.46-1.53)	.56
Cardiomyopathy	23	1	4.3	0.83 (0.12-5.64)	0.68 (0.10-4.61)	.70

Abbreviations: ACHD, adult congenital heart disease; AHD, acquired heart disease; CHD, congenital heart disease; CTD, conotruncal defects; LVOTO, left ventricular outflow tract obstruction; PDA, patent ductus arteriosus; PFO, patent foramen ovale; RHD, rheumatic heart disease; RR, risk ratio; RVOTO, right ventricular outflow tract obstruction; VHD, valvular heart disease.

^a^ Offspring without mothers with ACHD or AHD was used as the reference group. CHD incidence in this population was 5.2% (780 of 14 868).

^b^ Adjusted for maternal sociodemographic characteristics (age at conception, educational level, and occupation), reproductive factors (nulliparity, multiple gestations, in vitro fertilization and embryo transfer, reproductive history of stillbirth or congenital malformations, and abortion history), pregnancy complications (hypertensive disorders, diabetes, kidney disease, anemia, and prepregnancy overweight), and periconceptional exposures (smoking, alcohol use, folic acid supplementation, unstable emotions, and medication use).

^c^ False discovery rate *Q* < .05.

^d^ Nonrheumatic valvular anomaly.

### Maternal Cardiac Complications and Fetal Interventions

No maternal death occurred in our cohort. Cardiac complications were common in both maternal ACHD and AHD. Mothers with ACHD compared with those with AHD had a higher rate of prior cardiac surgery (200 of 368 [54.3%] vs 121 of 441 [27.4%]; *P* < .001) and arrhythmia (101 [27.4%] vs 102 [23.1%]; *P* < .001) (eTable 6 in Supplement 1). Most AHD surgeries (96 of 136 [70.6%]) involved valve prostheses. During follow-up, 5 fetuses with severe pulmonary stenosis or pulmonary atresia underwent in utero pulmonary valvuloplasty. All of them survived, and detailed information was provided previously.^25^

### Perinatal Adverse Outcomes in Offspring With CHD

Preterm birth had higher rates among offspring with CHD and mothers with ACHD as well as offspring with CHD and mothers without ACHD compared with offspring without CHD and mothers without cardiac disease (12 of 39 [30.8%] and 121 of 780 [15.5%] vs 1287 of 14 088 [9.1%]; all *P* < .001) (eTable 7 in Supplement 1). Higher rates of chromosomal (5 [12.8%] and 38 [4.9%] vs 75 [0.5%]; all with *P* < .001) and genetic aberrations (3 [7.7%] and 16 [2.1%] vs 57 [0.4%]; all with *P* < .001) were also observed among offspring with CHD and mothers with AHD as well as offspring with CHD and mothers without AHD compared with offspring without CHD and mothers without cardiac disease.

### Effect Modifiers

Associations between maternal ACHD or AHD and offspring CHD had higher effect sizes among primiparous women (ACHD: RR, 2.15 [95% CI, 1.48-3.11], *P* for interaction < .001; AHD: RR, 1.73 [95% CI, 1.17-2.56], *P* for interaction = .02) and women with periconceptional exposure to hazardous substances (ACHD: RR, 2.22 [95% CI, 1.56-3.16], *P* for interaction < .001; AHD: RR, 1.57 [95% CI, 1.05-2.36], *P* for interaction = .02) (Table 3). Additional modifiers for the maternal ACHD and offspring CHD association included multiple gestations (RR, 2.75; 95% CI, 1.33-5.68; *P* for interaction < .001), hypertensive disorders (RR, 3.98; 95% CI, 1.65-9.60; *P* for interaction = .03), unstable emotions (RR, 3.78; 95% CI, 1.68-8.47; *P* for interaction < .001), contraceptive medication use (RR, 2.86; 95% CI, 1.24-6.59; *P* for interaction = .005), and residence within 50 meters of a major roadway (RR, 2.11; 95% CI, 1.54-2.89; *P* for interaction < .001).

**Table 3.** Relative Risk of Offspring Congenital Heart Disease by Maternal Factors^a^

Maternal characteristic	Offspring						
	Without maternal ACHD or AHD [reference] (n = 14 868), case No./total No. (%)	With maternal ACHD (n = 368)			With maternal AHD (n = 441)		
		Case No./total No. (%)	Adjusted RR (95% CI)^b^	*P* value for interaction	Case No./total No. (%)	Adjusted RR (95% CI)^b^	*P* value for interaction
Sociodemographic							
Age, y							
≥35	175/3229 (5.4)	6/49 (12.2)	1.83 (0.85-3.95)	.27	11/107 (10.3)	1.66 (0.93-2.96)	.15
<35	605/11 637 (5.2)	33/319 (10.3)	1.67 (1.20-2.31)		28/333 (8.4)	1.25 (0.87-1.80)	
Educational level, y^c^							
≤12	239/2865 (8.3)	12/141 (8.5)	1.01 (0.58-1.76)	.55	13/155 (8.4)	0.94 (0.55-1.61)	.72
>12	539/11 993 (4.5)	27/227 (11.9)	2.49 (1.73-3.58)		26/286 (9.1)	1.80 (1.23-2.61)	
Household income per person per mo, ¥							
<3500	126/21 40 (5.9)	6/86 (7.0)	1.01 (0.46-2.22)	.99	5/82 (6.1)	0.77 (0.33-1.83)	.89
≥3500	654/12 728 (5.1)	33/282 (11.7)	1.96 (1.41-2.72)		34/359 (9.5)	1.54 (1.11-2.14)	
Occupation							
Unemployed or individual proprietor	247/2800 (8.8)	13/124 (10.5)	1.13 (0.67-1.91)	.20	16/153 (10.5)	1.07 (0.66-1.73)	.19
Other^d^	533/12 068 (4.4)	26/244 (10.7)	2.31 (1.59-3.36)		23/288 (8.0)	1.71 (1.14-2.55)	
Migrant^e^							
Yes	98/1698 (5.8)	5/52 (9.6)	1.26 (0.54-2.95)	.30	5/64 (7.8)	1.15 (0.48-2.73)	.10
No	682/13 170 (5.2)	34/316 (10.8)	1.78 (1.29-2.46)		34/377 (9.0)	1.43 (1.03-1.98)	
Reproductive factors							
Nulliparity							
Yes	441/9529 (4.6)	26/234 (11.1)	2.15 (1.48-3.11)	<.001	24/249 (9.6)	1.73 (1.17-2.56)	.02
No	339/5339 (6.3)	13/134 (9.7)	1.17 (0.70-1.98)		15/192 (7.8)	1.03 (0.63-1.70)	
Multiple gestations							
Yes	63/663 (9.5)	6/23 (26.1)	2.75 (1.33-5.68)	<.001	2/19 (10.5)	1.11 (0.29-4.20)	.75
No	717/14 205 (5.0)	33/345 (9.6)	1.65 (1.18-2.30)		37/422 (8.8)	1.43 (1.04-1.96)	
IVF-ET							
Yes	62/676 (9.2)	2/11 (18.2)	2.06 (0.54-7.90)	.31	2/25 (8.0)	0.80 (0.21-3.08)	.82
No	718/14 192 (5.1)	37/357 (10.4)	1.73 (1.27-2.37)		37/416 (8.9)	1.50 (1.09-2.05)	
Elective abortion history							
Yes	245/3954 (6.2)	9/94 (9.6)	1.21 (0.64-2.27)	.47	12/142 (8.5)	1.12 (0.64-1.94)	.50
No	535/10 914 (4.9)	30/274 (10.9)	2.02 (1.42-2.86)		27/299 (9.0)	1.53 (1.06-2.21)	
Spontaneous abortion history							
Yes	85/1246 (6.8)	3/45 (6.7)	0.81 (0.27-2.45)	.96	2/49 (4.1)	0.50 (0.13-1.99)	.39
No	695/13 622 (5.2)	36/323 (11.1)	1.86 (1.35-2.53)		37/392 (9.4)	1.52 (1.11-2.08)	
Pregnancy complications							
Hypertensive disorders							
Yes	36/739 (4.9)	5/25 (20.0)	3.98 (1.65-9.60)	.03	4/27 (14.8)	3.18 (1.19-8.46)	.17
No	744/14 129 (5.3)	34/343 (9.9)	1.61 (1.17-2.23)		35/414 (8.5)	1.31 (0.95-1.81)	
Diabetes^f^							
Yes	172/2773 (6.2)	4/60 (6.7)	0.87 (0.33-2.26)	.86	8/72 (11.1)	1.38 (0.70-2.72)	.25
No	608/12 095 (5.0)	35/308 (11.4)	1.97 (1.43-2.71)		31/369 (8.4)	1.39 (0.99-1.97)	
Kidney disease							
Yes	21/292 (7.2)	2/8 (25.0)	3.48 (0.98-12.37)	.08	3/22 (13.6)	1.90 (0.61-5.87)	.36
No	759/14 576 (5.2)	37/360 (10.3)	1.68 (1.23-2.29)		36/419 (8.6)	1.38 (1.01-1.90)	
Anemia							
Yes	69/939 (7.3)	2/31 (6.5)	0.76 (0.20-2.95)	.91	4/47 (8.5)	0.84 (0.32-2.21)	.73
No	711/13 929 (5.1)	37/337 (11.0)	1.86 (1.36-2.54)		35/394 (8.9)	1.45 (1.05-2.00)	
Prepregnancy overweight							
Yes	111/1741 (6.4)	2/33 (6.1)	0.72 (0.19-2.77)	.69	6/45 (11.1)	1.27 (0.55-2.92)	.40
No	669/13 127 (5.1)	37/335 (11.0)	1.88 (1.38-2.57)		34/396 (8.6)	1.38 (1.00-1.92)	
Self-reported periconceptional exposures^g^							
Passive smoking^h^							
Yes	219/3899 (5.6)	9/101 (8.9)	1.35 (0.72-2.54)	.34	11/107 (10.3)	1.47 (0.83-2.63)	.11
No	561/10 969 (5.1)	30/267 (11.2)	1.88 (1.33-2.66)		28/334 (8.4)	1.34 (0.93-1.93)	
Folic acid supplementation							
Yes	708/13 773 (5.1)	36/337 (10.7)	1.77 (1.29-2.42)	.06	35/404 (8.7)	1.38 (1.00-1.91)	.07
No	72/1095 (6.6)	3/31 (9.7)	1.20 (0.40-3.61)		4/37 (10.8)	1.30 (0.51-3.35)	
Unstable emotions							
Yes	41/508 (8.1)	5/18 (27.8)	3.78 (1.68-8.47)	<.001	1/16 (6.3)	0.57 (0.08-3.93)	.89
No	739/14 360 (5.1)	34/350 (9.7)	1.59 (1.15-2.20)		38/425 (8.9)	1.43 (1.05-1.95)	
Virus infection^i^							
Yes	200/3445 (5.8)	8/99 (8.1)	1.17 (0.60-2.30)	.46	12/105 (11.4)	1.55 (0.90-2.68)	.04
No	580/11 423 (5.1)	31/269 (11.5)	1.92 (1.37-2.69)		27/336 (8.0)	1.32 (0.91-1.91)	
Contraception medication use							
Yes	56/1008 (5.6)	5/28 (17.9)	2.86 (1.24-6.59)	.005	2/19 (10.5)	1.44 (0.36-5.70)	.61
No	724/13 860 (5.2)	34/340 (10.0)	1.61 (1.17-2.23)		37/422 (8.8)	1.39 (1.01-1.90)	
Other medication use^j^							
Yes	341/5363 (6.4)	21/176 (11.9)	1.61 (1.06-2.44)	.30	21/245 (8.6)	1.11 (0.73-1.70)	.26
No	439/9505 (4.6)	18/192 (9.4)	1.71 (1.09-2.68)		18/196 (9.2)	1.69 (1.08-2.65)	
Living in rooms newly renovated within 6 mo							
Yes	59/1107 (5.3)	1/14 (7.1)	1.48 (0.22-9.88)	.67	2/19 (10.5)	1.75 (0.46-6.62)	.44
No	721/13 761 (5.2)	38/354 (10.7)	1.74 (1.28-2.37)		37/422 (8.8)	1.38 (1.01-1.89)	
Residential proximity to main roadway: <50 m							
Yes	558/10 724 (5.2)	35/266 (13.2)	2.11 (1.54-2.89)	<.001	28/321 (8.7)	1.36 (0.95-1.95)	.09
No	222/4144 (5.4)	4/102 (3.9)	0.65 (0.25-1.73)		11/120 (9.2)	1.38 (0.77-2.47)	
Hazardous substances contact^k^							
Yes	441/8464 (5.2)	28/202 (13.9)	2.22 (1.56-3.16)	<.001	22/223 (9.9)	1.57 (1.05-2.36)	.02
No	339/6404 (5.3)	11/166 (6.6)	1.07 (0.60-1.91)		17/218 (7.8)	1.21 (0.76-1.94)	

Abbreviations: ACHD, adult congenital heart disease; AHD, acquired heart disease; CHD, congenital heart disease; IVF-ET, in vitro fertilization and embryo transfer.

SI conversion factor: To convert yuan to US dollars, multiply by 6.95.

^a^ Factors with a positive number of 1 or fewer in both the ACHD and AHD groups were deleted from the table, including ethnic minority identity, reproductive history of stillbirth or congenital malformations, periconceptional smoking, alcohol use, residential proximity to waste disposal stations or chemical plants (<1 km), and fever.

^b^ Adjusted for maternal sociodemographic characteristics (age at conception, educational level, and occupation), reproductive factors (nulliparity, multiple gestations, IVF-ET, reproductive history of stillbirth or congenital malformations, and abortion history), pregnancy complications (hypertensive disorders, diabetes, kidney disease, anemia, and prepregnancy overweight), and periconceptional exposures (smoking, alcohol use, folic acid supplementation, unstable emotions, and medication use). When analyzing the association of maternal ACHD and AHD with offspring CHD across the stratification of maternal factors, the corresponding factor was excluded from the adjustments.

^c^ There were 10 missing values for educational level in the reference group of offspring without mothers with ACHD or AHD.

^d^ Other occupation includes occupations beyond the categories shown here.

^e^ Migrants are defined as people living and working outside of their country of origin.

^f^ Diabetes includes pregestational and gestational, type 1 and type 2 diabetes.

^g^ Periconceptional period spans 6 months before conception through the first trimester.

^h^ Passive smoking is defined as exposure to tobacco smoke at home, workplace, or both.

^i^ Virus infection includes influenza, mumps, measles, rubella, chicken pox, hepatitis, or other infections.

^j^ Medication use includes use of Chinese medication or pharmaceutical chemicals except for contraception medication.

^k^ Hazardous substances contact is defined as exposure to noise, organic solvents, pesticides, paint, heavy metals, radiation, or other teratogens.

## Discussion

In this large, prospective birth cohort study from a high-volume cardiac referral center, both maternal ACHD and AHD were associated with increased risks of offspring CHD (eFigure 3 in Supplement 1). Minor CHDs, particularly septal defects, had the highest effect sizes, and maternal RVOTO and VHD emerged as key contributors to CHD risk. Furthermore, maternal ACHD was associated with higher risk of preterm birth among offspring with CHD, whereas maternal AHD was associated with elevated chromosomal and genetic abnormalities. The associations between maternal cardiac diseases and offspring CHD risk were amplified among primiparous women and women with periconceptional exposure to hazardous substances.

### Maternal ACHD and Offspring CHD

Our findings support prior evidence from high-income regions that maternal ACHD is associated with increased offspring CHD risk and extend this knowledge to a resource-limited setting.^16,18,19^ Unlike Western studies, which highlighted left ventricular outflow tract obstruction or Tetralogy of Fallot as the top ACHD subtypes for offspring CHD, the present study identified RVOTO as the highest risk factor.^14,16,19,26^ This discrepancy may reflect ethnicity variations in CHD recurrence patterns, as RVOTO is more common and left ventricular outflow tract obstruction occurs less frequently in Asian populations.^1^ No specific clustering of CHD phenotypes in relation to maternal ACHD subtypes was observed in our study, consistent with prior work showing no distinct familial aggregation beyond the general population.^27^ The association between ACHD and isolated CHD suggests single-gene inheritance,^28^ but most individuals with CHD represent sporadic cases.^29^ Our findings of minor CHDs, specifically septal defects, were more frequent in the offspring of mothers with ACHD. This result carries clinical importance given the increasing prevalence of minor CHDs, partly attributed to improved prenatal diagnosis and elective termination of critical CHDs.^30^

ACHD was also associated with preterm birth among offspring with CHD. Both maternal ACHD and fetal CHD are independent risk factors for adverse outcomes, yet management guidelines for this dual burden are lacking.^10,11,31,32,33^ At our center, a multidisciplinary team individualizes delivery planning, with cesarean delivery often chosen for maternal hemodynamic instability or fetal distress, which may also have contributed to increased preterm births.

### Maternal AHD and Offspring CHD

AHD was more frequent than ACHD in our cohort, reflecting the profile of low-income regions where AHD is the most common cardiac condition in pregnancy.^34^ In contrast, ACHD predominates in high-income settings due to improved survival of patients with CHD into childbearing age.^35^ For instance, in the Registry of Pregnancy and Cardiac Disease (ROPAC), which primarily enrolled pregnancies with cardiac diseases from high-income regions, ACHD accounted for 57% of the diseases compared with 43% for AHD.^12^ Our findings suggest that rheumatic heart disease remains common among pregnant women in China despite an overall decline.^36^ Meanwhile, the growing burden of other VHD lesions highlights the need for greater clinical attention to these conditions.

Maternal AHD was associated with increased risk of offspring CHD, with VHD as the highest risk factor. To our knowledge, ours is the first prospective study to quantify this risk. Our findings are supported by the New York SPARCS (Statewide Planning and Research Cooperative System) data showing higher CHD prevalence among offspring of mothers with AHD (4.7%), particularly VHD (3.0%), compared with the general population (1.3%).^20^ Maternal AHD was attributed to CHD associated with chromosomal or genetic aberrations, which was supported by both subtype and outcome analyses. This finding suggests that maternal AHD may predispose to broader developmental anomalies beyond isolated CHD, potentially through shared genetic risk or intrauterine perturbations.^37^

### Cardiac Complications and Effect Modifiers

Cardiac complications were common in both ACHD and AHD groups, with a higher rate of arrhythmia compared with prior evidence.^12,26,34,38^ This discrepancy may be attributable to our inclusion of minor arrhythmias, whereas prior studies typically included severe arrhythmias requiring intervention. Arrhythmia was also more common in pregnant women with ACHD than those with AHD, likely resulting from higher rates of prior surgeries in this group.^39^

Primiparity and periconceptional hazardous substance exposure amplified the association between maternal cardiac disease and offspring CHD. Compared with multiparous women, primiparous women may have more challenges in adapting to pregnancy-related physiological changes, particularly with underlying cardiac conditions.^40^ Hazardous substance exposure has been associated with fetal CHD.^41^ Our findings highlight that such exposure may exacerbate maternal cardiac outcomes. In addition, other effect modifiers were observed for maternal ACHD and offspring CHD associations. These are identified risk factors for fetal CHD and may act as additional stressors in pregnancies with ACHD.^42,43,44,45,46^

### Potential Mechanisms

Maternal ACHD may affect offspring CHD risk through both genetic transmission and intrauterine factors.^47^ Although most CHDs are sporadic,^29^ the higher recurrence of CHD in offspring of mothers with ACHD supports a strong genetic contribution,^28^ with autosomal dominant sequence variations reported in selected familial CHD phenotypes.^29,48,49,50,51,52^ Additionally, intrauterine conditions, such as altered hemodynamics, hypoxemia, chronic inflammation, and epigenetic regulation, may play a role.^53^

Maternal AHD may also induce offspring CHD by altering the intrauterine milieu. Maternal AHD affected both the immediate CHD and cardiovascular health later in life of the offspring through mechanisms such as the thrifty phenotype hypothesis and vascular remodeling.^13^ The suboptimal intrauterine environment may disrupt cardiac embryogenesis or exacerbate genomic instability, possibly explaining the role of AHD in associated CHD. These mechanisms require further investigation.

### Clinical Implications

Our findings highlight the need for early echocardiographic screening in offspring of mothers with cardiac disease, particularly RVOTO and VHD. Minor CHDs, especially septal defects, warrant careful attention. The association between maternal AHD and associated CHD supports expanded genetic counseling and advanced prenatal diagnosis. Both ACHD and AHD in pregnancy carry substantial maternal cardiac risks, underscoring the importance of multidisciplinary care at tertiary centers. Tailored antenatal surveillance and delivery planning are essential. Preparedness for preterm delivery should be emphasized for pregnant women with ACHD.

Given that parity and environmental exposures are effect modifiers, special attention should be paid to primiparous women and those at risk of hazardous exposures. Preventive counseling for reproductive-aged women with cardiac disease may help reduce the burden of CHD in the next generation.

### Strengths and Limitations

This study has several strengths. First, to our knowledge, this study is one of the largest prospective birth cohorts to assess maternal cardiac disease and CHD risk. All pregnant women at a cardiac referral center over 10 years were included, which, when coupled with a high participation rate, resulted in a well-characterized cohort. Second, structured and systematic screening for CHD in offspring was conducted, extending to 1 year of age to enable the detection of late-presenting cases. This approach minimized the possibility of missed diagnoses of CHD. Third, in addition to the occurrence of CHD, we also assessed the association of maternal ACHD and AHD with the outcomes of offspring CHD, providing a broader clinical perspective. Fourth, comprehensive confounding variables were adjusted for, enhancing the internal validity of this study.

However, several limitations should be acknowledged. First, as a single-center study at a high-resource referral center, generalizability may be limited. The observed higher baseline incidence likely reflects more sensitive detection algorithms^8^; inclusion of terminations, fetal losses, and multiple gestations with higher CHD rates^42,54,55^; and extended follow-up to 1 year of age with late-presenting cases captured. Nevertheless, sensitivity analyses excluding referrals and minor cases yielded consistent results. Second, despite the large overall sample, stratification by specific CHD phenotypes and adverse outcomes yielded small subgroup sizes, making these results exploratory. Third, although we included a wide range of covariates, residual confounding by unmeasured parental genetic or epigenetic factors or specific medications cannot be ruled out. Fourth, multiple comparisons increase the risk of false-positives despite statistical correction through the use of false discovery rates. Finally, the absence of systematic long-term postnatal assessment constrains the interpretation of later-life health implications, and the genetic abnormalities could not be mapped to specific chromosomal loci due to insufficient genetic data.

## Conclusions

In this cohort study of pregnant women and their offspring, maternal ACHD and AHD were associated with increased risks of offspring CHD and adverse outcomes. Minor CHDs, particularly septal defects, were most commonly associated with maternal cardiac disease. RVOTO and VHD were key factors in offspring CHD risk. Maternal factors that modify the associations between maternal cardiac disease and offspring CHD highlighted opportunities for targeted surveillance and intervention. These findings support further investigation into intergenerational cardiovascular health in high-risk women as well as efforts to elucidate the exact chromosomal loci associated with the detected genetic abnormalities.

## Notes

Supplement 1. **eMethods 1.** Offspring CHD Diagnosis and Classification **eFigure 1.** Schematic Representation of CHD Screening and Diagnosis **eMethods 2.** Definition of Confounders and Potential Effect Modifiers **eFigure 2.** Flow Chart of Study Participant Inclusion **eTable 1.** Maternal Baseline Characteristics of Offspring With and Without Maternal ACHD/AHD, n (%) **eTable 2.** Specific Diagnoses of Maternal ACHD and AHD **eTable 3.** Specific CHD Phenotypes Diagnosed in Fetuses **eTable 4.** Risk Ratios of CHD in Offspring With Maternal ACHD and AHD **eTable 5.** Risk Ratios (RR) of Overall CHD in Offspring With Maternal ACHD and AHD, Results of the Sensitivity Analyses **eTable 6.** Cardiac Complications in Pregnant Women With ACHD and AHD **eTable 7.** Independent and Joint Effects of Maternal ACHD/AHD and Offspring CHD on Other Adverse Birth Outcomes **eFigure 3.** Maternal Cardiac Disease and Risk of Congenital Heart Disease in Offspring

Supplement 2.
Data Sharing Statement
