# Supplementary material for: Maternal Cardiac Disease and Congenital Heart Disease Risk in Offspring
Source: JAMA Netw Open. 2026 May 5;9(5):e2610823. doi: 10.1001/jamanetworkopen.2026.10823 (PMC13147197; doi:10.1001/jamanetworkopen.2026.10823)
Supplement: Supplement 1. — eMethods 1. Offspring CHD Diagnosis and Classification eFigure 1. Schematic Representation of CHD Screening and Diagnosis eMethods 2. Definition of Confounders and Potential Effect Modifiers eFigure 2. Flow Chart of Study Participant Inclusion eTable 1. Maternal Baseline Characteristics of Offspring With and Without Maternal ACHD/AHD, n (%) eTable 2. Specific Diagnoses of Maternal ACHD and AHD eTable 3. Specific CHD Phenotypes Diagnosed in Fetuses eTable 4. Risk Ratios of CHD in Offspring With Maternal ACHD and AHD eTable 5. Risk Ratios (RR) of Overall CHD in Offspring With Maternal ACHD and AHD, Results of the Sensitivity Analyses eTable 6. Cardiac Complications in Pregnant Women With ACHD and AHD eTable 7. Independent and Joint Effects of Maternal ACHD/AHD and Offspring CHD on Other Adverse Birth Outcomes eFigure 3. Maternal Cardiac Disease and Risk of Congenital Heart Disease in Offspring [file jamanetwopen-e2610823-s001.pdf]

Supplemental Online Content

Qu Y, Liu X, Lin S, et al. Maternal cardiac disease and congenital heart disease risk in offspring. *JAMA Netw Open*. 2026;9(5):e2610823. doi:10.1001/jamanetworkopen.2026.10823

eMethods 1. Offspring CHD Diagnosis and Classification

eFigure 1. Schematic Representation of CHD Screening and Diagnosis

eMethods 2. Definition of Confounders and Potential Effect Modifiers

eFigure 2. Flow Chart of Study Participant Inclusion

eTable 1. Maternal Baseline Characteristics of Offspring With and Without Maternal ACHD/AHD, n (%)

eTable 2. Specific Diagnoses of Maternal ACHD and AHD

eTable 3. Specific CHD Phenotypes Diagnosed in Fetuses

eTable 4. Risk Ratios of CHD in Offspring With Maternal ACHD and AHD

eTable 5. Risk Ratios (RR) of Overall CHD in Offspring With Maternal ACHD and AHD, Results of the Sensitivity Analyses

eTable 6. Cardiac Complications in Pregnant Women With ACHD and AHD

eTable 7. Independent and Joint Effects of Maternal ACHD/AHD and Offspring CHD on Other Adverse Birth Outcomes

eFigure 3. Maternal Cardiac Disease and Risk of Congenital Heart Disease in Offspring

This supplemental material has been provided by the authors to give readers additional information about their work.

© 2026 Qu Y et al. *JAMA Network Open*

eMethods 1. Offspring CHD diagnosis and classification

Offspring CHD was diagnosed and confirmed through a comprehensive multi-stage approach during pregnancy and after birth. This approach involves fetal ultrasound screening, fetal echocardiographic examination, neonatal cardiac evaluations, postnatal echocardiography confirmation, and follow-up assessments of the offspring. In detail, all pregnancies underwent basic ultrasound screening for fetal cardiac anomalies between 18 to 24 weeks of gestation. Suspected CHD fetuses were further evaluated through echocardiography at 22 to 26 weeks of gestation to confirm the diagnosis. Following delivery, according to international guidelines, each newborn received a clinical cardiac assessment before discharge, usually within 72 hours.[1] Newborns with suspected CHD and those prenatally diagnosed underwent postnatal echocardiography to confirm the CHD diagnoses. Live births were followed up until one year after birth to identify any late-identifying CHD. When available, CHD diagnoses were further corroborated through additional methods, such as autopsy, chromosomal microarray analysis, genetic test, computed tomography, cardiac catheterization, and surgery. Each CHD case was reviewed by two senior pediatric cardiologists, and a third one resolved any disagreements.

All CHD phenotypes were coded using the International Classification of Diseases, Tenth Revision (ICD-10) (Q20.000–Q28.000). CHD phenotypes were first classified as “associated CHD” or “isolated CHD” depending on the presence or absence of chromosomal/genetic aberration and accompanying non-cardiac defects. CHDs were then grouped into categories according to the plurality of CHD lesions, such as “multiple CHD” if at least two CHD phenotypes were present, or “single CHD” if only one CHD phenotype was present. One predominant phenotype with the most severe hemodynamic pathology was assigned to each case with multiple CHD diagnoses. CHDs were further categorized according to the severity as “critical CHD” if prenatal structural malformations of the heart were present that usually require intervention during the first year of life or as “minor CHD” [including atrial septal defect (ASD), ventricular septal defect (VSD), patent ductus arteriosus (PDA), and patent foramen

© 2026 Qu Y et al. *JAMA Network Open*

ovale (PFO)].[2] CHD phenotypes were finally categorized into main categories based on etiology as follows: conotruncal defects, atrioventricular septal defect (AVSD), anomalous pulmonary venous return (APVR), left ventricular outflow tract obstruction (LVOTO), right ventricular outflow tract obstruction (RVOTO), septal defect, single ventricle, PFO or PDA, and other specified CHD defects.[3] To obtain strong statistical power, we grouped all the phenotypes into septal and non-septal defects when detecting the association between maternal ACHD and AHD and offspring CHD phenotypes.

1. Advisory Committee on Heritable Disorders in Newborns and Children (Febrary 2024). Recommended Uniform Screening Panel. https://www.hrsa.gov/advisory-committees/heritable-disorders. .
2. Liberman, R.F., *et al.* Delayed diagnosis of critical congenital heart defects: trends and associated factors. *Pediatrics* **134**, e373-381 (2014).
3. Qu, Y., *et al.* First-Trimester Maternal Folic Acid Supplementation Reduced Risks of Severe and Most Congenital Heart Diseases in Offspring: A Large Case-Control Study. *J Am Heart Assoc* **9**, e015652 (2020).

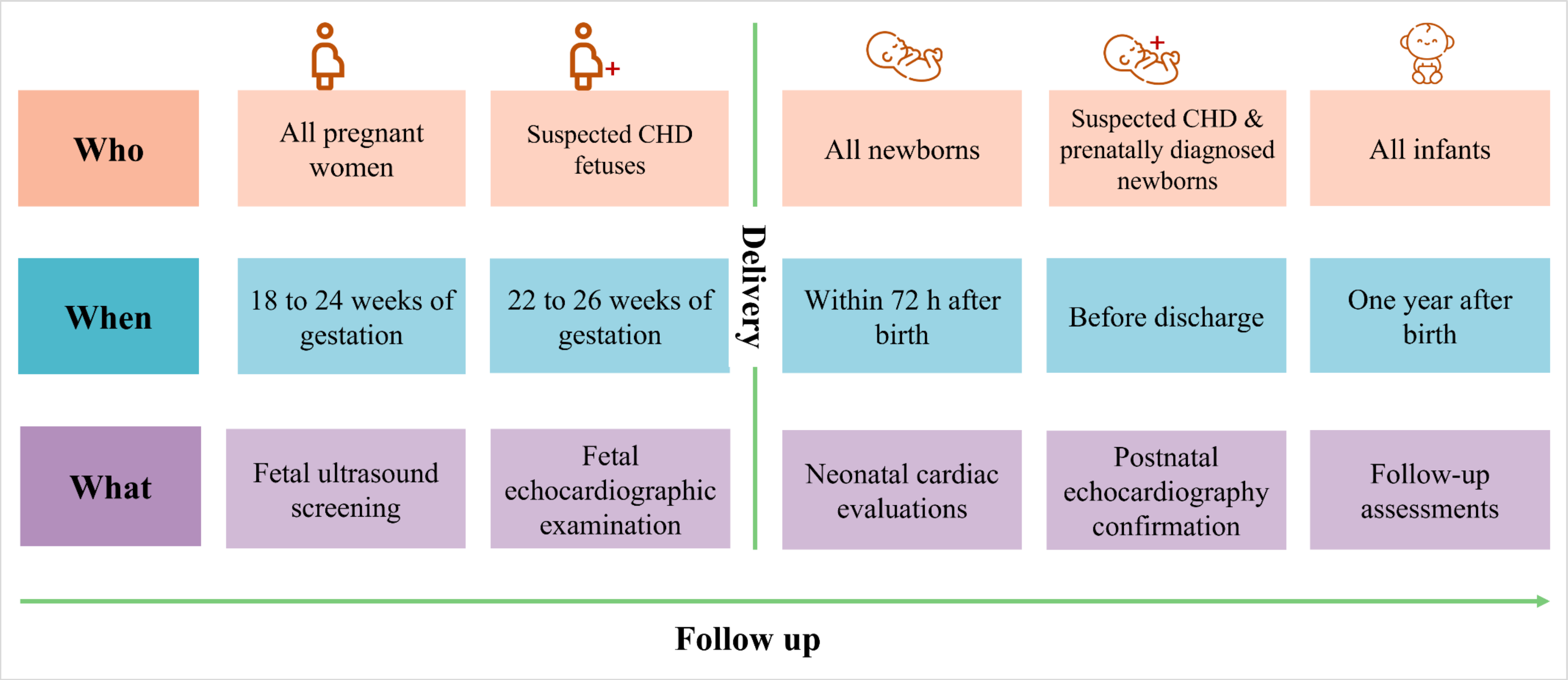

eFigure 1. Schematic representation of CHD screening and diagnosis

© 2026 Qu Y et al. *JAMA Network Open*

eMethods 2. Definition of confounders and potential effect modifiers

1. Confounders

Maternal sociodemographic characteristics:

- Age at conception (continuous)
- Education (categorized: >12 years, ≤12 years)
- Occupation (categorized: unemployment or individual proprietor, others)

Reproductive factors:

- Nulliparity (yes/no)
- Multiple gestations (yes/no, pregnancy with more than one fetus, i.e., twins, triplets+)
- In vitro fertilization and embryo transfer (IVF-ET, yes/no)
- Prior pregnancy with stillbirth or congenital malformations (yes/no)
- Abortion history (yes/no)

Pregnancy complications:

- Hypertensive disorders (yes/no, 140/90 mmHg on two occasions)
  - Preeclampsia (hypertension criteria plus .0.3 g proteinuria in the 24 h urine sample)
  - Eclampsia (pre-eclampsia with grand mal seizures)
- Diabetes (yes/no, ADA diagnostic criteria, type I, type II, and gestational diabetes mellitus)
- Renal diseases (yes/no, chronic kidney diseases or pregnancy-induced kidney diseases)
- Anemia (yes/no, hemoglobin <110 g/L or hematocrit < 33%)
- Prepregnant overweight (yes/no, preconception body mass index ≥ 24 kg/m^2 according to the Chinese guidelines)

Periconceptional behaviors and exposures

- Smoking (yes/no, on average consume at least one cigarette per day)
- Alcohol drinking (yes/no, alcohol intake of on average at least 50 ml/d without specifying wine)

© 2026 Qu Y et al. *JAMA Network Open*

- Folic acid supplementation (yes/no, taking at least 0.4 mg of folic acid daily for >5 days per week continuously)
- Unstable emotion (yes/no)
- Medicine use (yes/no)

2. Potential effect modifier definitions

Maternal sociodemographic characteristics:

- Age at conception (categorized: <35 years, ≥35 years)
- Education (categorized: >12 years, ≤12 years)
- Household income (categorized: <3500, ≥3500 per person per month, CNY)
- Occupation (categorized: unemployment or individual proprietor, others)
- Migrants (yes/no, people living and working outside their origin)

Reproductive factors:

- Nulliparity (yes/no)
- Multiple gestations (yes/no, pregnancy with more than one fetus, i.e., twins, triplets+)
- In vitro fertilization and embryo transfer (IVF-ET, yes/no)
- Elective abortion history (yes/no)
- Spontaneous abortion history (yes/no)

Pregnancy complications:

- Hypertensive disorders (yes/no, 140/90 mmHg on two occasions)
- Diabetes (yes/no, ADA diagnostic criteria, including type I, type II, and gestational diabetes mellitus)
- Renal diseases (yes/no, chronic kidney diseases or pregnancy-induced kidney diseases)
- Anemia (yes/no, hemoglobin <110 g/L or hematocrit < 33%)
- Prepregnant overweight (yes/no, preconception body mass index ≥ 24 kg/m^2 according to the Chinese guidelines)

Periconceptional behaviors and exposures

- Passive smoking (yes/no, exposure to tobacco smoke at home, workplace, or both)

© 2026 Qu Y et al. *JAMA Network Open*

- Folic acid supplementation (yes/no, taking at least 0.4 mg of folic acid daily for >5 days per week continuously)
- Unstable emotion (yes/no)
- Virus infection (yes/no, infection of influenza, mumps, measles, rubella, chicken pox, hepatitis, or others)
- Contraception medicine use (yes/no)
- Other medicine use (yes/no, usage of Chinese medicine or pharmaceutical chemicals except for contraception medicine)
- Living in rooms newly renovated within 6 months (yes/no)
- Residential proximity to the main roadway (<50 m) (yes/no)
- Hazardous substances contact (yes/no, exposure to noise, organic solvents, pesticides, paint, heavy metals, radiation, or other teratogens)

Maternal sociodemographic characteristics and periconceptional behaviors and exposures were self-reported during the face-to-face interview and captured through a structured questionnaire. Pregnancy complications and reproductive factors were extracted from the electronic medical record system using a standardized case report form and double-checked during the face-to-face interviews for detailed information. The periconceptional period in the current study refers to six months before conception until enrollment.

© 2026 Qu Y et al. *JAMA Network Open*

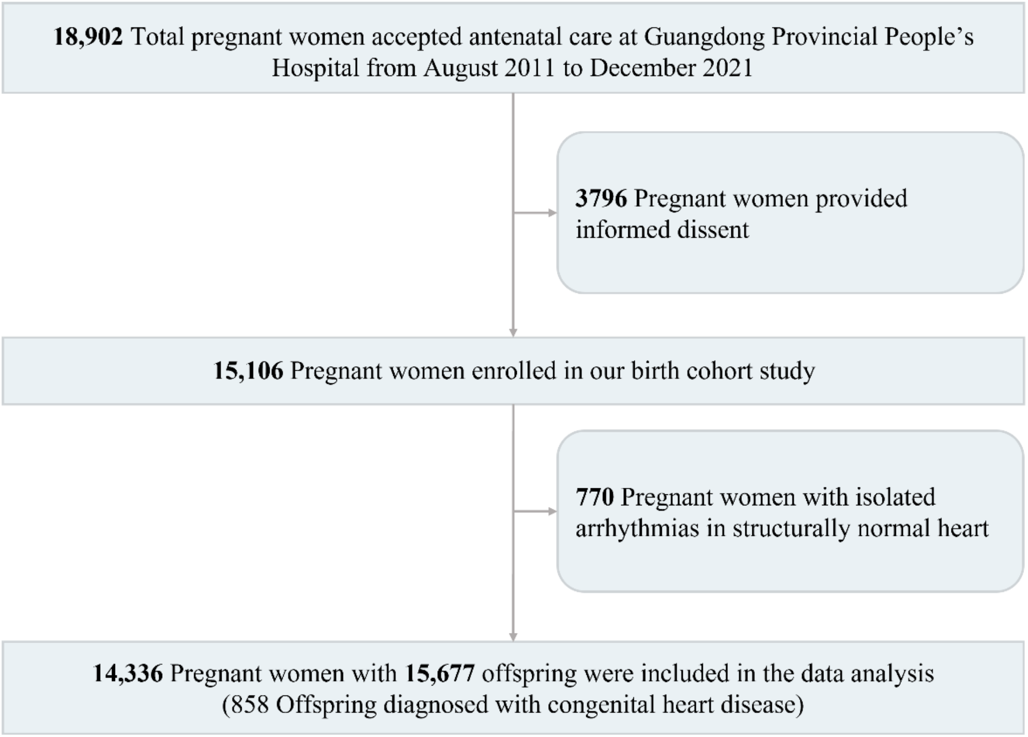

eFigure 2. Flow chart of study participant inclusion

© 2026 Qu Y et al. *JAMA Network Open*

eTable 1. Maternal baseline characteristics of offspring with and without maternal ACHD/AHD, n (%)

Maternal characteristics	Offspring, No. (%)				*P*-value
	Overall (n = 15,677)	Without ACHD/AHD (n = 14868)	With maternal ACHD (n = 368)	With maternal AHD (n = 441)	
**Diagnosis of offspring CHD**					
Yes	858	780 (5.2)	39 (10.6)	39 (8.8)	<0.001
No	14819	14088 (94.8)	329 (89.4)	402 (91.2)	
**Maternal demographics**					
Age, mean (SD), y	31.4±4.5	31.4±4.5	29.8±4.6	31.7±4.6	<0.001
Education[a]					
High school and less	1402	1230 (8.3)	73 (19.8)	99 (22.4)	<0.001
Senior high school	1759	1635 (11.0)	68 (18.5)	56 (12.7)	
College	10859	10385 (69.9)	216 (58.7)	258 (58.5)	
Master and above	1647	1608 (10.8)	11 (3.0)	28 (6.3)	
Household income per person per month, CNY					
< 3500	2308	2140 (14.4)	86 (23.4)	82 (18.6)	<0.001
≥ 3500	13369	12728 (85.6)	282 (76.6)	359 (81.4)	
Occupation					
Unemployment	1577	1408 (9.5)	75 (20.4)	94 (21.3)	<0.001
Individual proprietor	1500	1392 (9.4)	49 (13.3)	59 (13.4)	
Industry worker	711	665 (4.5)	21 (5.7)	25 (5.7)	
Managerial and commercial service	4712	4499 (30.3)	107 (29.1)	106 (24.0)	
Government servant and professional	6487	6255 (42.1)	96 (26.1)	136 (30.8)	
Others	690	649 (4.4)	20 (5.4)	21 (4.8)	
Ethnicity					
Han	15339	14545 (97.8)	361 (98.1)	433 (98.2)	0.83
Minorities	338	323 (2.2)	7 (1.9)	8 (1.8)	
Migrants[b]					
Yes	1814	1698 (11.4)	52 (14.1)	64 (14.5)	0.04
No	13863	13170 (88.6)	316 (85.9)	377 (85.5)	
**Maternal reproductive factors**					
Nulliparity					
Yes	10012	9529 (64.1)	234 (63.6)	249 (56.5)	0.004
No	5665	5339 (35.9)	134 (36.4)	192 (43.5)	
Reproductive history of stillbirth/congenital malformations					
Yes	419	394 (2.6)	7 (1.9)	18 (4.1)	0.12
No	15258	14474 (97.4)	361 (98.1)	423 (95.9)	

© 2026 Qu Y et al. *JAMA Network Open*

Elective abortion history					
Yes	4190	3954 (26.6)	94 (25.5)	142 (32.2)	0.03
No	11487	10914 (73.4)	274 (74.5)	299 (67.8)	
Spontaneous abortion history					
Yes	1340	1246 (8.4)	45 (12.2)	49 (11.1)	0.005
No	14337	13622 (91.6)	323 (87.8)	392 (88.9)	
Multiple gestations					
Yes	705	663 (4.5)	23 (6.3)	19 (4.3)	0.26
No	14972	14205 (95.5)	345 (93.8)	422 (95.7)	
IVF-ET					
Yes	712	676 (4.5)	11 (3.0)	25 (5.7)	0.19
No	14965	14192 (95.5)	357 (97.0)	416 (94.3)	
Pregnancy complications					
Hypertensive disorders					
Yes	791	739 (5.0)	25 (6.8)	27 (6.1)	0.17
No	14886	14129 (95.0)	343 (93.2)	414 (93.9)	
Diabetes[c]					
Yes	2905	2773 (18.7)	60 (16.3)	72 (16.3)	0.25
No	12772	12095 (81.3)	308 (83.7)	369 (83.7)	
Renal disease					
Yes	322	292 (2.0)	8 (2.2)	22 (5.0)	<0.001
No	15355	14576 (98.0)	360 (97.8)	419 (95.0)	
Anemia					
Yes	1017	939 (6.3)	31 (8.4)	47 (10.7)	<0.001
No	14660	13929 (93.7)	337 (91.6)	394 (89.3)	
Prepregnant overweight					
Yes	1819	1741 (11.7)	33 (9.0)	45 (10.2)	0.17
No	13858	13127 (88.3)	335 (91.0)	396 (89.8)	
Maternal self-reported periconceptional exposures[d]					
Smoking[e]					
Yes	108	103 (0.7)	2 (0.5)	3 (0.7)	0.94
No	15569	14765 (99.3)	366 (99.5)	438 (99.3)	
Passive smoking[f]					
Yes	4107	3899 (26.2)	101 (27.4)	107 (24.3)	0.56
No	11570	10969 (73.8)	267 (72.6)	334 (75.7)	
Alcohol drinking[g]					
Yes	105	102 (0.7)	0	3 (0.7)	0.28
No	15572	14766 (99.3)	368 (100)	438 (99.3)	
Folic acid supplementation					
Yes	14514	13773 (92.6)	337 (91.6)	404 (91.6)	0.55
No	1163	1095 (7.4)	31 (8.4)	37 (8.4)	
Unstable emotion					
Yes	542	508 (3.4)	18 (4.9)	16 (3.6)	0.30

© 2026 Qu Y et al. *JAMA Network Open*

No	15135	14360 (96.6)	350 (95.1)	425 (96.4)	
Virus infection[h]					
Yes	3649	3445 (23.2)	99 (26.9)	105 (23.8)	0.24
No	12028	11423 (76.8)	269 (73.1)	336 (76.2)	
Fever (>38.5 °C)					
Yes	1096	1045 (7.0)	26 (7.1)	25 (5.7)	0.54
No	14581	13823 (93.0)	342 (92.9)	416 (94.3)	
Contraception medication use					
Yes	1055	1008 (6.8)	28 (7.6)	19 (4.3)	0.10
No	14622	13860 (93.2)	340 (92.4)	422 (95.7)	
Other medication use[i]					
Yes	5784	5363 (36.1)	176 (47.8)	245 (55.6)	<0.001
No	9893	9505 (63.9)	192 (52.2)	196 (44.4)	
Living in rooms newly renovated within 6 months					
Yes	1140	1107 (7.4)	14 (3.8)	19 (4.3)	0.002
No	14537	13761 (92.6)	354 (96.2)	422 (95.7)	
Residential proximity to main roadway (<50 m)					
Yes	11311	10724 (72.1)	266 (72.3)	321 (72.8)	0.95
No	4366	4144 (27.9)	102 (27.7)	120 (27.2)	
Hazardous substances contact[j]					
Yes	8889	8464 (56.9)	202 (54.9)	223 (50.6)	0.02
No	6788	6404 (43.1)	166 (45.1)	218 (49.4)	
Residential proximity to waste disposal stations or chemical plants (<1 km)					
Yes	268	251 (1.7)	9 (2.4)	8 (1.8)	0.53
No	15409	14617 (98.3)	359 (97.6)	433 (98.2)	

Abbreviations: ACHD, adult congenital heart disease; AHD, acquired heart disease; CHD, congenital heart disease; CNY, Chinese Yuan; IVF-ET, in vitro fertilization & embryo transfer; N, number; SD, standard deviation.

[a] There were 10 missing values of maternal education;

[b] Migrants: people living and working outside their origin;

[c] Diabetes: includes pregestational and gestational, type 1 and type 2 diabetes;

[d] Periconceptional period: spans from six months before conception until the review;

[e] Smoking: consuming on average at least one cigarette per day;

[f] Passive smoking: exposure to tobacco smoke at home, workplace, or both;

[g] Alcohol drinking: intake of on average at least 50 ml of alcohol per day.

[h] Virus infection: infection of influenza, mumps, measles, rubella, chicken pox, hepatitis, or others;

[i] Medication use: usage of Chinese medication or pharmaceutical chemicals except for contraception medication;

[j] Hazardous substances contact: exposure to noise, organic solvents, pesticides, paint, heavy metals, radiation, or other teratogens.

© 2026 Qu Y et al. *JAMA Network Open*

eTable 2. Specific diagnoses of maternal ACHD and AHD

Maternal cardiac disease diagnoses	Frequency	Proportion (%)
Total	809	100
ACHD	368	45.5
CTD	27	7.3
TGA	5	1.4
TOF	21	5.7
DORV	1	0.3
AVSD	13	3.5
APVR	2	0.5
LVOTO	14	3.8
CoA	1	0.3
A(v)S	9	2.4
Mitral valve stenosis	4	1.1
RVOTO	32	8.7
Ebstein anomaly	13	3.5
P(v)S	14	3.8
PA	1	0.3
Isolated RVOTO	4	1.1
Septal defects	207	56.3
ASD	135	36.7
VSD	72	19.6
PFO/PDA	40	10.9
Congenital valve heart disease	13	3.5
Marfan syndrome	6	1.6
Other specified ACHDs	14	3.8
AHD	441	54.5
VHD	209	47.4
RHD	173	39.2
Cardiomyopathy	23	5.2
IE	8	1.8
Other specified AHDs	28	6.3

Abbreviations: ACHD, adult congenital heart disease; AHD, acquired heart disease; APVR, anomalous pulmonary venous return; A(v)S, aortic (valve) stenosis; ASD, atrial septal defect; AVSD, atrioventricular septal defect; CHD, congenital heart disease; CoA, coarctation of the aorta; CTD, conotruncal defects; DORV, double-outlet right ventricle; IAA, interrupted aortic arch; IE, infective endocarditis; LVOTO, left ventricular outflow tract obstruction; N, number; PA, pulmonary atresia; PDA, patent ductus arteriosus; PFO, patent foramen ovale; P(v)S, pulmonary (valve) stenosis; RHD, rheumatic heart disease; RVOTO, right ventricular outflow tract obstruction; TGA, transposition of the great aorta; TOF, tetralogy of Fallot; VHD, valvular heart disease, excluding rheumatic valvular anomaly; VSD, ventricular septal defect.

© 2026 Qu Y et al. *JAMA Network Open*

eTable 3. Specific CHD phenotypes diagnosed in fetuses

Fetal CHD diagnoses	Frequency	Proportion (%)
Total	858	100
By the coexistence of chromosomal, genetic, or non-cardiac abnormalities		
Isolated CHD	756	88.1
Associated CHD	102	11.9
By the plurality of CHD lesions		
Single CHD	298	34.7
Multiple CHD	560	65.3
By the severity of CHD lesions		
Minor CHD	449	52.3
Critical CHD	409	47.7
By the cause of CHD lesions		
CTD	124	14.5
TGA	74	8.6
TOF	35	4.1
DORV	14	1.6
Truncus arteriosus	1	0.1
AVSD	9	1.0
APVR	16	1.9
LVOTO	45	5.2
IAA	6	0.7
CoA	38	4.4
A(v)S	1	0.1
RVOTO	61	7.1
Ebstein anomaly	3	0.3
P(v)S	44	5.1
PA	14	1.6
Septal defects	345	40.2
ASD	220	25.6
VSD	49	5.7
VSD+ASD	76	8.9
SV	4	0.5
PFO/PDA	104	12.1
Other specified CHDs	150	17.5

Abbreviations: APVR, anomalous pulmonary venous return; A(v)S, aortic (valve) stenosis; ASD, atrial septal defect; AVSD, atrioventricular septal defect; CHD, congenital heart disease; CoA, coarctation of the aorta; CTD, conotruncal defects; DORV, double-outlet right ventricle; IAA, interrupted aortic arch; LVOTO, left ventricular outflow tract obstruction; N, number; PA, pulmonary atresia; PDA, patent ductus arteriosus; PFO, patent foramen ovale; P(v)S, pulmonary (valve) stenosis; RVOTO, right ventricular outflow tract obstruction; SV, single ventricle; TGA, transposition of the great aorta; TOF, tetralogy of fallot; VSD, ventricular septal defect.

© 2026 Qu Y et al. *JAMA Network Open*

eTable 4. Risk ratios of CHD in offspring with maternal ACHD and AHD

Fetal CHD types	Maternal absence of ACHD and AHD (Reference)	Maternal ACHD			Maternal AHD		
	Case n / total n (%)	Case n / total n (%)	RR-Model 1[a]	RR-Model 2[b]	Case n / total n (%)	RR-Model 1[a]	RR-Model 2[b]
**Overall CHDs**	**780/14868 (5.2)**	**39/368 (10.6)**	**2.02 (1.49-2.74)**	**1.71 (1.26-2.31)**	**39/441 (8.8)**	**1.69 (1.24-2.29)**	**1.38 (1.02-1.87)**
Isolated CHDs	691/14779 (4.7)	35/364 (9.6)	2.06 (1.49-2.84)	1.74 (1.26-2.40)	30/432 (6.9)	1.49 (1.04-2.11)	1.23 (0.87-1.75)
Associated CHDs[c]	89/14177 (0.6)	4/333 (1.2)	1.91 (0.71-5.18)	1.71 (0.63-4.66)	9/411 (2.2)	3.49 (1.77-6.88)	2.64 (1.33-5.25)
Single CHDs	271/14359 (1.9)	14/343 (4.1)	2.16 (1.28-3.66)	2.12 (1.25-3.61)	13/415 (3.1)	1.66 (0.96-2.87)	1.57 (0.91-2.73)
Multiple CHDs	509/14597 (3.5)	25/354 (7.1)	2.03 (1.38-2.98)	1.60 (1.09-2.35)	26/428 (6.1)	1.74 (1.19-2.55)	1.32 (0.90-1.93)
Minor CHDs	393/14481 (2.7)	28/357 (7.8)	2.89 (2.00-4.18)	2.62 (1.81-3.79)	28/430 (6.5)	2.40 (1.66-3.48)	2.14 (1.47-3.11)
Critical CHDs	387/14475 (2.7)	11/340 (3.2)	1.21 (0.67-2.18)	0.96 (0.54-1.73)	11/413 (2.7)	1.00 (0.55-1.80)	0.74 (0.41-1.34)
Septal defects	298/14386 (2.1)	24/353 (6.8)	3.28 (2.20-4.91)	2.95 (1.97-4.43)	23/425 (5.4)	2.61 (1.73-3.95)	2.28 (1.50-3.45)
Non-septal defects	482/14570 (3.3)	15/344 (4.4)	1.32 (0.80-2.18)	1.08 (0.66-1.79)	16/418 (3.8)	1.16 (0.71-1.89)	0.91 (0.56-1.48)

Abbreviations: ACHD, adult congenital heart disease; AHD, acquired heart disease; CHD, congenital heart disease; N, number; RR, risk ratio.

[a] Model 1 is crude.

[b] Model 2 adjusted for maternal demographics (age at conception, education, and occupation), reproductive factors (nulliparity, multiple gestations, in vitro fertilization & embryo transfer, reproductive history of stillbirth or congenital malformations, and abortion history), pregnancy complications (hypertensive disorders, diabetes, renal disease, anemia, and prepregnant overweight), and periconceptional behaviors (smoking, alcohol drinking, folic acid supplementation, unstable emotion, and medicine use);

[c] Associated CHDs: CHD associated with chromosomal or genetic abnormalities, or non-cardiac defects.

© 2026 Qu Y et al. *JAMA Network Open*

eTable 5. Risk ratios (RR) of overall CHD in offspring with maternal ACHD and AHD, results of the sensitivity analyses

Sensitivity analyses	Maternal absence of ACHD and AHD (Reference)	Maternal ACHD			Maternal AHD		
	CHD offspring n / total n (%)	CHD offspring n / total n (%)	RR-Model 1[a]	RR-Model 2[b]	CHD offspring n / total n (%)	RR-Model 1[a]	RR-Model 2[b]
Excluding participants referred from other hospitals	213/6374 (3.3)	7/61 (11.5)	3.43 (1.69-6.98)	3.45 (1.69-7.01)	8/76 (10.5)	3.15 (1.61-6.15)	3.08 (1.57-6.01)
Only including singletons	717/14205 (5.0)	33/345 (9.6)	1.90 (1.36-2.64)	1.65 (1.18-2.30)	37/422 (8.8)	1.74 (1.27-2.38)	1.43 (1.04-1.96)
Only including singletons from primiparous women	408/9055 (4.5)	22/221 (10.0)	2.21 (1.47-3.32)	2.15 (1.44-3.20)	23/238 (9.7)	2.03 (1.35-3.05)	1.80 (1.21-2.69)
Excluding PFO	720/14868 (4.8)	37/368 (10.1)	2.08 (1.52-2.84)	2.04 (1.50-2.79)	35/441 (7.9)	1.64 (1.18-2.27)	1.51 (1.09-2.08)

Abbreviations: ACHD, adult congenital heart disease; AHD, acquired heart disease; ARSA, aberrant right subclavian artery; CHD, congenital heart disease; N, number; PFO, patent foramen ovale; PLSVC, persistent left superior vena cava; RR, risk ratio.

[a] Model 1 is crude;

[b] Model 2 adjusted for maternal demographics (age at conception, education, and occupation), reproductive factors (nulliparity, multiple gestations, in vitro fertilization & embryo transfer, reproductive history of stillbirth or congenital malformations, and abortion history), pregnancy complications (hypertensive disorders, diabetes, renal disease, and anemia), and periconceptional behaviors (prepregnant overweight, smoking, alcohol drinking, folic acid supplementation, unstable emotion, and medicine use).

© 2026 Qu Y et al. *JAMA Network Open*

eTable 6. Cardiac complications in pregnant women with ACHD and AHD

Maternal cardiac complications	Offspring, No. (%)		P-value[a]
	With maternal ACHD (n=368)	With maternal AHD (n=441)	
Cardiac surgery	208 (56.5)	136 (30.8)	<0.001
Before pregnancy	200 (54.3)	121 (27.4)	<0.001
During pregnancy	8 (2.2)	15 (3.4)	0.30
Hypertensive disorders	25 (6.8)	27 (6.1)	0.70
Preeclampsia or eclampsia	4 (1.1)	4 (0.9)	0.85
Arrhythmia	101 (27.4)	102 (23.1)	<0.001
Pulmonary hypertension	33 (9.0)	46 (10.4)	0.49
Signs of heart failure (NYHA ≥ II)	269 (73.1)	318 (72.1)	0.75
Post-partum hemorrhage	5 (1.4)	4 (0.9)	0.54

Abbreviations: ACHD, adult congenital heart disease; AHD, acquired heart disease, NYHA, the New York Heart Association.
[a] Comparison between mothers with ACHD and those with AHD.

© 2026 Qu Y et al. *JAMA Network Open*

eTable 7. Independent and joint effects of maternal ACHD/AHD and offspring CHD on other adverse birth outcomes*

Fetal outcomes	Offspring, No. (%)†					Offspring, No. (%)†				
	Offspring CHD-, maternal cardiac disease- (n=14088)	Offspring CHD-, maternal ACHD+ (n=329)	Offspring CHD+, maternal ACHD- (n=780)	Offspring CHD+, maternal ACHD+ (n=39)	Overall *P*-value‡	Offspring CHD-, maternal cardiac disease- (n=14088)	Offspring CHD-, maternal AHD+ (n=402)	Offspring CHD+, maternal AHD- (n=780)	Offspring CHD+, maternal AHD+ (n=39)	Overall *P*-value‡
Pregnancy outcomes										
Live births	13992 (99.3)$_{a}$	325 (98.8)$_{a}$	725 (92.9)$_{b}$	39 (100)$_{a, b}$	<0.001	13992 (99.3)$_{a}$	395 (98.3)$_{a, b}$	725 (92.9)$_{c}$	36 (92.3)$_{b, c}$	<0.001
Termination	51 (0.4)$_{a}$	1 (0.3)$_{a}$	31 (4.0)$_{b}$	0$_{a, b}$		51 (0.4)$_{a}$	3 (0.7)$_{a, b}$	31 (4.0)$_{c}$	2 (5.1)$_{b, c}$	
Fetal death	45 (0.3)$_{a}$	3 (0.9)$_{a}$	3 (0.4)$_{a}$	0$_{a}$		45 (0.3)$_{a}$	4 (1.0)$_{a}$	3 (0.4)$_{a}$	0$_{a}$	
Neonatal death	0$_{a}$	0$_{a}$	21 (2.7)$_{b}$	0$_{a, b}$		0$_{a}$	0$_{a}$	21 (2.7)$_{b}$	1 (2.6)$_{b}$	
FGR	230 (1.6)$_{a}$	5 (1.5)$_{a}$	15 (1.9)$_{a}$	2 (5.1)$_{a}$	0.35	230 (1.6)$_{a}$	6 (1.5)$_{a, b}$	15 (1.9)$_{a, b}$	3 (7.7)$_{b}$	0.03
SGA	81 (0.6)$_{a}$	0$_{a}$	4 (0.5)$_{a}$	0$_{a}$	0.54	81 (0.6)$_{a}$	3 (0.7)$_{a}$	4 (0.5)$_{a}$	0$_{a}$	0.92
Preterm birth	1287 (9.1)$_{a}$	60 (18.2)$_{b}$	121 (15.5)$_{b}$	12 (30.8)$_{c}$	<0.001	1287 (9.1)$_{a}$	84 (20.9)$_{b}$	121 (15.5)$_{b}$	9 (23.1)$_{b}$	<0.001
Low birth weight	903 (6.5)$_{a}$	37 (11.4)$_{b}$	100 (13.4)$_{b, c}$	11 (28.2)$_{c}$	<0.001	903 (6.5)$_{a}$	55 (13.9)$_{b}$	100 (13.4)$_{b}$	7 (18.9)$_{b}$	<0.001
Chromosomal aberration	75 (0.5)$_{a}$	4 (1.2)$_{a}$	38 (4.9)$_{b}$	1 (2.6)$_{a, b}$	<0.001	75 (0.5)$_{a}$	5 (1.2)$_{a}$	38 (4.9)$_{b}$	5 (12.8)$_{c}$	<0.001
Genetic aberration	57 (0.4)$_{a}$	4 (1.2)$_{a, b}$	16 (2.1)$_{b}$	0$_{a, b}$	<0.001	57 (0.4)$_{a}$	3 (0.7)$_{a, b}$	16 (2.1)$_{b}$	3 (7.7)$_{c}$	<0.001
Non-cardiac defects	174 (1.2)$_{a}$	2 (0.6)$_{a}$	39 (5.0)$_{b}$	3 (7.7)$_{b}$	<0.001	174 (1.2)$_{a}$	12 (3.0)$_{b}$	39 (5.0)$_{b}$	2 (5.1)$_{a, b}$	<0.001

Abbreviations: ACHD, adult congenital heart disease; AHD, acquired heart disease; CHD, congenital heart disease; FGR, fetal growth restriction; SGA, small for gestational age.

* Different subscript letters indicate statistically significant differences between groups with P<0.05;

† "+" means positive and "-" means negative;

‡ Overall *P*-values reflect the results of the chi-square tests.

© 2026 Qu Y et al. *JAMA Network Open*

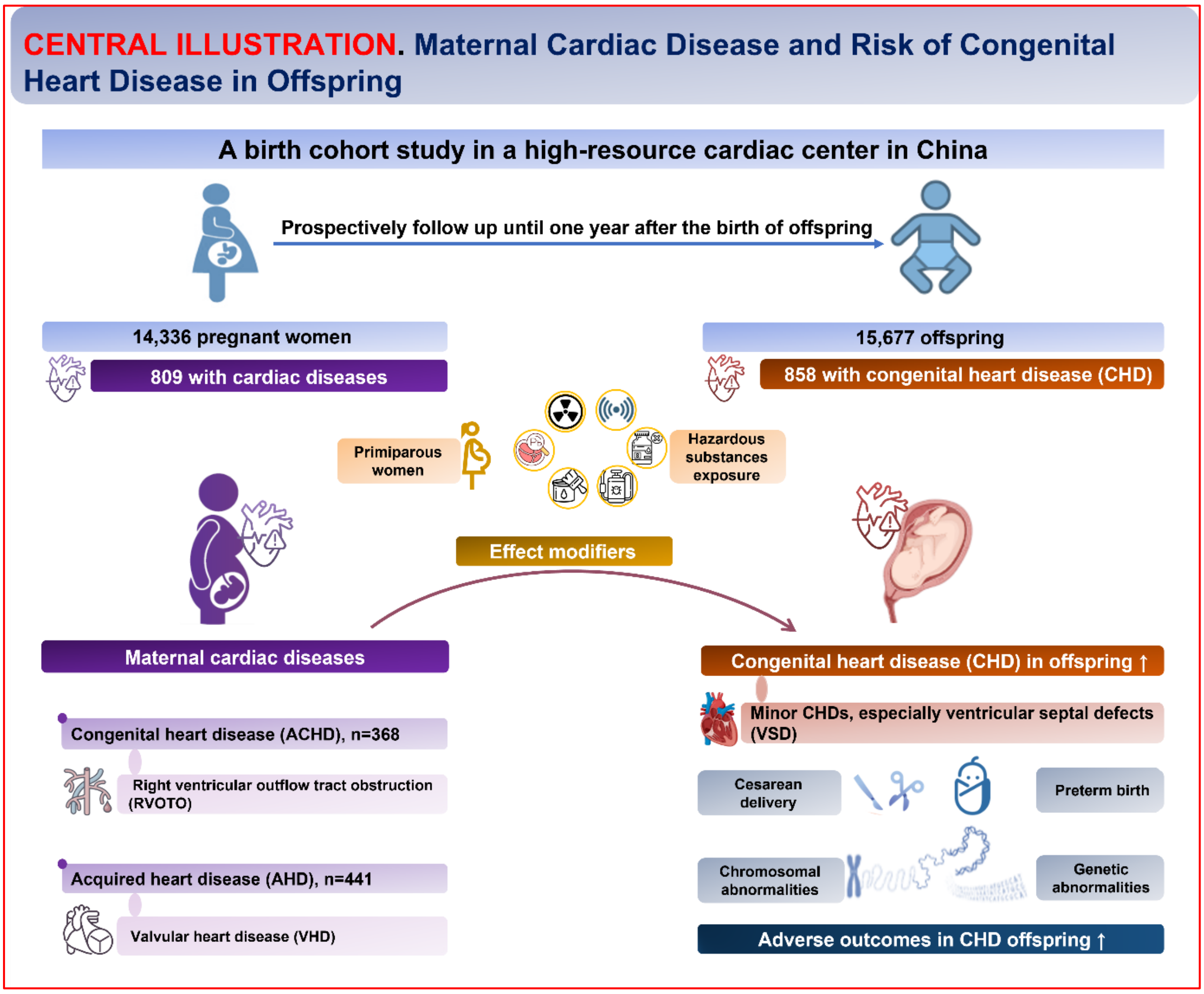

eFigure 3. Maternal cardiac disease and risk of congenital heart disease in offspring

In this 10-year birth cohort study of 14,336 pregnant women and 15,677 offspring from a major cardiac referral center in China, both maternal ACHD and AHD were associated with significantly increased risks of offspring CHD, particularly septal defects. Right ventricular outflow tract obstruction (RVOTO) and valvular heart disease (VHD) were key maternal cardiac contributors to offspring CHD risk. Among CHD offspring, maternal ACHD was linked to more preterm births, while maternal AHD was linked to increased chromosomal and genetic abnormalities. These associations were notably stronger in primiparous women and those with periconceptional exposure to environmental hazards.

© 2026 Qu Y et al. *JAMA Network Open*
