# Supplementary material for: Maternal Cardiac Disease and Congenital Heart Disease Risk in Offspring
Source: JAMA Netw Open. 2026 May 5;9(5):e2610823. doi: 10.1001/jamanetworkopen.2026.10823 (PMC13147197; doi:10.1001/jamanetworkopen.2026.10823)
Supplement: Supplement 2. — Data Sharing Statement [file jamanetwopen-e2610823-s002.pdf]

Data Sharing Statement

Qu. Maternal Cardiac Disease and Congenital Heart Disease Risk in Offspring. *JAMA Netw Open*. Published May 05, 2026. doi:10.1001/jamanetworkopen.2026.10823

Data
Data available: Yes
Data types: Deidentified participant data
How to access data: The data will be available upon reasonable request to the corresponding author at jimei_1965@outlook.com for academic use only.
When available: With publication

Supporting Documents
Document types: None

Additional Information
Who can access the data: Researchers whose proposed use of the data has been approved
Types of analyses: For academic use only.
Mechanisms of data availability: After approval of a proposal.
